# Automatic classification of sentences to support Evidence Based Medicine

**DOI:** 10.1186/1471-2105-12-S2-S5

**Published:** 2011-03-29

**Authors:** Su Nam Kim, David Martinez, Lawrence Cavedon, Lars Yencken

**Affiliations:** 1 NICTA VRL, The University of Melbourne, 3010, Australia; 2 Department of Computer Science and Software Engineering, The University of Melbourne, 3010, Australia; 3 School of Computer Science and IT, RMIT University, Melbourne 3000, Australia

## Abstract

**Aim:**

Given a set of pre-defined medical categories used in Evidence Based Medicine, we aim to automatically annotate sentences in medical abstracts with these labels.

**Method:**

We constructed a corpus of 1,000 medical abstracts annotated by hand with specified medical categories (e.g. *Intervention*, *Outcome*). We explored the use of various features based on lexical, semantic, structural, and sequential information in the data, using Conditional Random Fields (CRF) for classification.

**Results:**

For the classification tasks over all labels, our systems achieved micro-averaged f-scores of 80.9% and 66.9% over datasets of structured and unstructured abstracts respectively, using sequential features. In labeling only the key sentences, our systems produced f-scores of 89.3% and 74.0% over structured and unstructured abstracts respectively, using the same sequential features. The results over an external dataset were lower (f-scores of 63.1% for all labels, and 83.8% for key sentences).

**Conclusions:**

Of the features we used, the best for classifying any given sentence in an abstract were based on unigrams, section headings, and sequential information from preceding sentences. These features resulted in improved performance over a simple bag-of-words approach, and outperformed feature sets used in previous work.

## Introduction

*Evidence Based Medicine* (*EBM*) is an approach to clinical practice whereby medical decisions are informed by primary evidence, such as the results of *randomized control trials* (*RCTs*). Evidence-based practice requires efficient information access to such evidence, and also retrieval and analysis of documents relevant to a specified clinical topic. Evidence-based practitioners use specific criteria when judging whether an RCT is relevant to a given question. They generally follow the *PICO* criterion [1]: *Population* (*P*) (i.e., participants in a study); *Intervention* (*I*); *Comparison* (*C*) (if appropriate); and *Outcome* (*O*) (of an Intervention). Variations and extensions of this classification have been proposed, such as the PECODR tagset [2]. To better serve the information needs of the EBM community, we explore the use of classification techniques to identify relevant key sentences in a given document, and classify these against specified medical criteria. Such information could be leveraged in various ways: e.g., to improve search performance; to enable structured querying with specific categories; and to aid users in more quickly making judgements against specified PICO criteria.

In this paper, we build a classifier that performs two tasks. First, it identifies the key sentences in an abstract, filtering out those that do not provide the most relevant information. Second, it classifies sentences according to medical tags (based on the PICO criteria) used by our medical research partners. We project these two tasks into an (*N*+1)-way classification task, with *N* semantic labels for key sentences and 1 label (i.e. *Other*) for labeling non-key sentences. For this purpose, we have built a corpus of 1,000 medical abstracts, hand-annotated at the sentence level by domain experts, which we use to develop and evaluate our system.

A major difference of our approach from previous work is the combination of key-sentence identification and classification, whereas others (e.g., [3,4]) have separated these tasks and assumed that all sentences are relevant at the classification step. Many sentences in abstracts do not fall into any of the pre-defined categories (due to vagueness, diversion from the central topic, etc.), and the identification of such extraneous material is useful.

Our classification techniques use an extensive set of features, derived from context, semantic relations, structure and sequencing of the text. In particular, for sequence information we use features from previous sentences in the given abstract, and use predicted labels as features in a novel way. We employ Conditional Random Fields (CRF) [5], which are well suited for learning over sequential data (such as cohesive, structured text). In the following sections, we first describe related work in Section . In Section , we provide details of our experimental setup including the construction of the corpus, the learners, and features. We present our results and error analysis in Sections and respectively. We conclude and discuss future directions in Section .

## Related work

The generalised use of PICO and similar schemas by clinicians when performing search, and their improvement on performance in user studies [6], has fueled interest in the development of automatic aids for this task.

Demner-Fushman and Lin [7] were the first to present automatic classifiers for PICO-elements. In their work, they used the MetaMap parser [8], hand-built rules, and statistical classifiers to identify sentences or phrases in abstracts relevant to each PICO element. Only for the element *Outcome* did they use a supervised classifier (Naive Bayes) with a large set of features, including n-grams, position, and semantic information from MetaMap. They trained this classifier over 275 hand-annotated abstracts, and reported accuracies in the range of 74%-93% depending on the type of abstract and the evaluation threshold. It is important to note that this is the only previous work in the literature that uses the Other tag as we do. Demner-Fushman and Lin [7] also applied their final PICO classifiers to a novel weighting formula for medical information retrieval (IR), significantly improving the baseline for the task. In a related paper [9], the same authors applied PICO classification to the task of clustering medical results, showing that it improved information delivery. The main limitations of their classifiers were the small size of the annotated data, and the reliance on hand-crafted rules for some of the PICO classes. One drawback of their IR system was the use of parameters that were hand-assigned or estimated over a small dataset. More recently, Chung [4] performed PICO classification by combining rhetorical roles with PICO elements, in order to achieve higher performance and alleviate the hand-annotation cost. Chung uses four rhetorical roles, namely *Aim*, *Method*, *Results*, and *Conclusions*; she requires that each sentence in an abstract fall into one of these roles. Chung then focuses on categorising one PICO class at a time, for a more fine-grained analysis. There are two limitations to this approach: (i) the overall classification performance across medical tags is not known; and (ii) sentences are forced to always have one semantic tag. We address these limitations by focusing directly on labels of interest for EBM, allowing sentences to be labeled as *Other*, and by allowing multiple labels per sentence when required. We believe that this is a more realistic setting than the one presented in previous work, and will provide better insight on the performance we should expect for this kind of task. This makes our approach not directly comparable to [4], but we are able to apply her technique and features and evaluate the performance of her system over medical labels only. We also extend her approach by adding new types of features.

Other work on sentence classification has focused on *rhetorical role classification*, which aims at identifying the roles of sentences in text (e.g. Motivation, Result, etc.). Training and test data for this task is easy to obtain from structured scientific abstracts, which provide section headings. This approach has been used in many supervised systems [3,10-13]. With respect to feature representations, previous work has relied mostly on contextual features, such as n-grams and words in specific locations. Heuristics derived from sequential features of abstracts, such as relative location of sentences and section headings have recently been explored [3,4]. In terms of finding suitable machine learners, well-known machine learning techniques have been applied to the tasks, including *Naïve Bayes* (NB) [7], *Support Vector Machines* (SVM) [4], *Hidden Markov Models* (HMM) [14], and *Conditional Random Fields* (CRF) [4]. Also, [12] proposed a probability-based learner inspired by the sequence of abstracts.

Recent work by [15] has shown the difficulty of identifying PICO elements in text, and has proposed a location-based information retrieval weighting strategy, motivated by the distribution of PICO elements. The authors also applied a weighting model based on the PICO information from the query, obtaining significant improvements from both approaches. However, their annotation of PICO tags was based on open text, disregarding sentence boundaries, which led to agreement problems between the annotators. Further, their classifier was built using the section headings of structured abstracts (e.g. *Patients*, *Outcome’*, etc.) without human supervision, which could introduce noise.

## Method

In this section we describe the construction of the corpus, the classifiers and features, and the experimental setting.

### Data collection

We extracted 1,000 abstracts from MEDLINE for annotation. Our focus was on medical research, and in order to extract relevant abstracts we used queries from two institutions that develop systematic reviews of the literature: The Global Evidence Mapping Initiative (GEM) [16], and The Agency for Healthcare Research and Quality (AHRQ) [17]. GEM focuses on traumatic brain injury and spinal cord injury, and they provided the results of hand-constructed queries targeting diverse aspects of this subdomain. We randomly extracted 500 abstracts from a list of 74,000 query results for our annotation.

In order to diversify the contents of the corpus, the remaining 500 abstracts were randomly sampled from a set of AHRQ queries covering different medical issues (e.g. “Systematic Review of the Literature Regarding the Diagnosis of Sleep Apnoea”).

**Figure 1:**
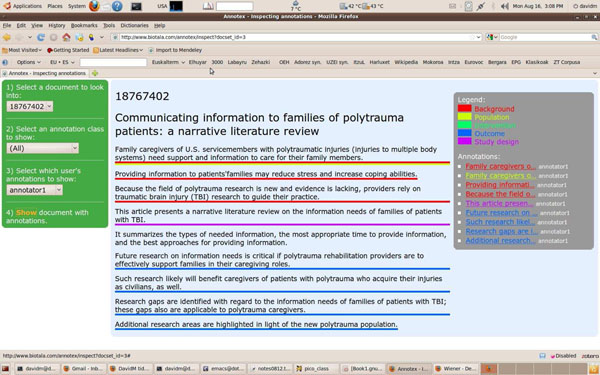
Annotex interface for the annotation of sentences.

Some of the abstracts used in our experiments (376 out of 1,000) are structured, which means that they contain section headings (e.g. *Aim*, *Method*, etc.). These headings are helpful in capturing the rhetorical structure of the text, and we use them as features (when available). See the abstract of this paper you are reading for an example of a *structured* abstract, and Figure 1 for an example of an *unstructured* abstract.

### Annotation

In order to define our tagset we first adopted the 7-way annotation scheme presented in [18]. (We thank the authors for kindly providing a sample of their data for our work, as well as initial definitions for semantic tags.) After analysing this data, we decided to drop two of their categories (“Statistics” and “Supposition”) because their work showed significant agreement problems on those classes. We also decided to add the category *Study Design*, based on feedback by medical experts at GEM on the utility of this category. Thus, our annotation categories are as follows:

• *Background*: Material that informs and may place the current study in perspective, e.g. work that preceded the current; information about disease prevalence; etc;

• *Population*: The group of individual persons, objects, or items comprising the study’s sample, or from which the sample was taken for statistical measurement;

• *Intervention*: The act of interfering with a condition to modify it or with a process to change its course (includes prevention);

• *Outcome*: The sentence(s) that best summarizes the consequences of an intervention;

• *Study Design*: The type of study that is described in the abstract;

• *Other*: Any sentence not falling into one of the other categories and presumed to provide little help with clinical decision making, i.e. non-key or irrelevant sentences.

The 1,000 abstracts were annotated by a medical student over 80 hours, with the continuous collaboration of a senior medical expert. Each sentence could be annotated with multiple classes. In order to make annotation easier, we built the “Annotex” tool, which provides an interface to the sentence-segmented corpus. A screenshot of the tool interface is shown in Figure 1.

In order to measure agreement, 60 of the abstracts were blindly annotated by one of the authors, and Cohen’s kappa was calculated. The original annotation was not changed in any case. The averaged score over all classes was 0.62, which indicates “substantial agreement” [19]. The kappa values for the different classes are given in Table 1. The table shows that most classes have good agreement scores, and only *Study Design* seems problematic. This annotated data is available for further research, and can be obtained by emailing the contact author.

**Table 1:** Kappa values per class.

Class	Kappa
Background	0.70
Intervention	0.61
Other	0.67
Outcome	0.71
Population	0.63
Study Design	0.41

### Conditional random fields

Our sentence-classifier uses Conditional Random Fields (CRFs) [5] for the learning algorithm. CRFs provide a discriminative framework for building structured models to segment and label sequence data. CRFs are undirected graphical models in which each vertex represents a random variable whose distribution is to be inferred, and each edge represents a dependency between two random variables. In our case the sentences in an abstract are represented by vertices, and the edges represent the relationship between sentences. CRFs have the advantage that they both model sequential effects and support the use of a large number of features; they have also been shown to perform comparatively well in other sentence-classification tasks [3,4].

In our implementation, we use the Mallet package [20], applying the Gaussian prior given in the default setting for all our experiments.

### Features

We trained our classifier with four sets of features that we describe in turn; some of these features are novel for this kind of task.

#### Lexical information

Collocational information, such as surrounding bag-of-words (BOW), is a simple and effective way to capture the semantic similarity between two texts. We extend this idea by also using bigrams, which consist of all consecutive pairs of terms present in the sentence. We also utilise the POS (Part-of-Speech) information of the tokens in the BOW and bigram representations—we used the CPAN module Lingua::EN::Tagger as our POS-tagger.

BOW features have been extensively used for sentence classification [3,10,11]. More specifically, [4] applied POS tags in the same way as we do. However bigrams have not previously been applied to this kind of task.

#### Semantic information

We extend our feature set by using the Metathesaurus from the *Unified Medical Language System* (*UMLS*) [21], which provides a set of ontologies for the biomedical domain with semantic relationships between terms (e.g. synonyms and hypernyms). We use this resource in two ways: (i) directly querying the thesaurus for each token in the input, and (ii) parsing each sentence with the MetaMap analyser [8]. As a result we obtain Concept Unique Identifiers (CUIs), which map the text into the ontological concepts. This allows us to identify connections between different word forms of the same concept. For instance, the terms “disease” and “disorder” are listed under the same CUI in the *UMLS*, and this connection is potentially useful for measuring text similarity.

We use the extracted CUIs to define our main semantic features: *Token-CUI* and *MetaMap-CUI*. For the token approach, we expand this representation by extracting the synonym list for each CUI. We then use these new terms directly, or broken down into single terms (in case of multiword terms). This last feature is motivated by [22], who showed improved document classification results after breaking down multiwords for partial matches. In summary, we use the following four types of semantic features:

• *Token-CUI*: Concept identifiers (CUIs) extracted from direct queries.

• *Token-Syn*: Synonyms of each token in the sentence.

• *Token-Syn-B*: Synonyms in break-down form for each token.

• *MetaMap-CUI*: CUIs extracted from MetaMap.

#### Structural information

Previous work has found that the position of a sentence in an abstract can be important for its semantic classification [3,4,11]. For example, we expect that sentences related to *Aim* or *Motivation* will tend to occur at the beginning of an abstract, while those related to *Result*, *Discussion* or *Conclusion* will appear closer to the end. Thus, one of our structural features reflects the position of sentences from the beginning of the abstract.

Our other structural feature is obtained from section headings (when available). Section heading capture the rhetorical structure of the text, with tags such as *Conclusions*. These headings split the abstract into thematic parts, and we rely on the heading tag as a feature to represent all the sentences below the given heading. We explore two types of heading-based features: (i) the heading string is used without modification; and (ii) we map each heading string into one of four main rhetorical roles — namely, *Aim*, *Method*, *Results*, *Conclusions* — which have been used previously [3,11]. We rely on regular expressions to identify section headings, and on manual mapping of the different forms into the four main roles. We apply the same high-precision regular expression as used in [3], and manually map the different types of headings into the four rhetorical roles. Apart from feature engineering, previous work has mapped section headings for the purpose of building annotated corpora [15,23]; however, we use these headings only for feature representation. Heading features are only available for structured abstracts.

#### Sequential information

Sequential features leverage dependencies between different sentences in the text. For example, sentences for a particular subtopic (e.g. *Background*) typically occur sequentially as a group, and do not tend to repeat in later context. In addition, a subtopic in the *i*th sentence can be understood by analysing subtopics in preceding sentences from the abstract.

In order to model these dependencies we designed two types of features: *direct* and *indirect* dependencies. *Direct dependencies* use the labels of previous sentences, which are obtained by relying on the CRF trained on other types of features. *Indirect dependency* features are simply obtained by attaching the features of previous sentences to the target one. Regarding the number of sentences, for direct features we explore the use of window-sizes of 1, 3, and *all* previous sentences; for indirect features we test the results with 1, 2, or 3 previous sentences.

Sequential features may seem redundant when using sequential classifiers, but previous work has demonstrated good performance for these features for related classification tasks. For example, [24] used indirect features for dialogue act classification, while [25] described a method for classifying semantic labels of posts in web forum data as well as determining the links between posts.

In the medical domain, previous classification work has applied indirect dependency features [3,4], but not direct dependency ones. To facilitate comparison with the results from [4], we will also experiment with *windowed features*, which are features drawn from the previous and following sentence.

### Experimental setting

For our experiments we split the corpus of 1,000 abstracts into two sets: structured abstracts (*S*) and unstructured abstracts (*U*). The statistics of these two sets are given in Table 2. We also distinguish between two types of classification tasks: (1) *6-way* to classify both key sentences with the semantic labels and non-key sentences with *Other*; and (2) *5-way* to label key sentences only. Most related work has ignored irrelevant sentences in abstracts, considering only those sentences mapped to categories of interest [3,4]; by performing *5-way* classification we can compare to some degree our performance to previous work.

**Table 2:** Number of abstracts and sentences for Structured (S) and Unstructured (U) abstract sets, including number of sentences per class.

	All	S	U
# Abstracts	1000	376	624

# Sentences	10379	4774	5605
- Background	2557	669	1888
- Intervention	690	313	377
- Outcome	4523	2240	2283
- Population	812	369	443
- Study Design	233	149	84
- Other	1564	1034	530

Thus, we have four groups of experiments. Note that in all performance tables over our dataset the results will be shown over these four groups. For each dataset we use 10-fold cross-validation, and measure micro-averaged precision, recall, and f-score. Precision is given for each class by the number of true positives divided by the total number of elements predicted as belonging to the class. To obtain recall we divide the true positives by the total number of elements that actually belong to the class in the test data. We calculate the micro-average for all classes by combining the results of each test instance, as opposed to averaging the results of the classes (macro-average). The f-score gives us the harmonic mean of precision and recall.

Finally, as an external corpus for evaluation, we use the small dataset from [18] (kindly provided by the authors), which consists of 100 abstracts (51 of them structured). As discussed in Section , this dataset uses a slightly different tagset to the one we have been using. Hence, for our final experiments, the classes “Statistics”, “Supposition”, and *Study Design* were mapped into *Other*.

## Results

In this section, we first evaluate the performance of a benchmark system, which uses features that have been previously explored in the literature. We then analyse the different feature sets described earlier (lexical, semantic, structural, and sequential) in turn. Finally, we evaluate our system over an external dataset.

### Benchmark system

As our benchmark system, we measure the performance of the system from [4] over our dataset. We were able to partially replicate that system by using the same tool and parameters (Mallet), and similar features. The features consist of word features (unigrams with their POS), positional information, section headings, and windowed features (features from the previous and following sentence). The difference from the experiments described in [4] is that we do not perform the term normalisation step, and we applied a different POS tagger.

The results given in Table 3 compare the use of different sets of features over our dataset. We can see that recall tends to be lower than precision, but the differences are not large: this is due to the fact that most target sentences have unique labels. We henceforth use f-score to compare the different approaches. Regarding the type of data, we see that classification over the structured abstracts clearly outperforms classification over unstructured ones. Even without using section headings, structured abstracts are better suited to our classification task. As we would expect, the results for 5-way classification are much better than for 6-way classification. Overall, the best results are obtained by using all the features for structured data, and ignoring windowed features for unstructured data. We will compare the rest of our results to the best benchmark configurations.

**Table 3:** F-scores for the benchmark system based on [4]. 1.P: unigrams with POS, Pst: position, W: windowed features, Sec: section headings. Best results per column are given in bold.

Feature	6-way						5-way					
		S			U			S			U		
	
		P	R	F	P	R	F	P	R	F	P	R	F
1.P+Pst	75.11	71.49	73.26	**66.24**	**61.93**	**64.01**	86.38	81.07	83.64	**73.63**	**68.33**	**70.88**
1.P+Pst+W	72.40	68.91	70.62	64.14	59.96	61.98	85.07	79.84	82.37	72.61	67.39	69.90
1.P+Pst+Sec+W	**79.45**	**75.62**	**77.48**	–	–	–	**90.37**	**84.81**	**87.50**	–	–	–

### Adding lexical and semantic information

We first evaluate the use of features independently in Table 4. The top section of the table presents the results of the lexical features, and we can see that unigrams perform better than bigrams, which suffer from data sparseness. The performance of semantic features (in the bottom section) is lower than for unigrams; the extra effort to extract these features does not pay off. The reasons for the low performances seem to be the sparseness of the terms found by token-querying, and the ambiguity in the MetaMap output. From our experiments we conclude that these semantic resources directly (without tuning or filtering) do not contribute positively to the task. Overall, the results using lexical and semantic features individually are lower than the benchmark.

**Table 4:** F-scores using lexical and semantic Information for 6-way and 5-way classification.

Feature	6-way		5-way	
		S	U	S	U
1.P	**70.42**	**60.82**	**81.68**	**68.51**
2.P	47.50	44.19	59.09	49.61
Token-CUI	66.19	59.47	78.26	65.57
Token-Syn	64.13	58.79	76.77	65.47
Token-Syn-B	65.25	59.94	77.43	66.22
MetaMap-CUI	56.08	52.23	64.58	56.58

1.P: unigrams with POS, 2.P: bigrams with POS, CUI: UMLS tag, Syn: expansion with synonyms, Syn-B: synonyms in break-down form. Best results per column are given in bold.

The performance for selected combinations of features are given in Table 5; we focus on combinations using unigrams with POS, which seems to provide the most robust configuration over these feature types. Overall, we do not see any significant performance improvements.

**Table 5:** F-scores of Combining Lexical and Semantic Information.

Feature	6-way		5-way	
		S	U	S	U
1.P+2.P	**67.87**	60.53	**81.10**	68.47
1.P+T-CUI	67.83	61.01	79.94	67.41
1.P+T-Syn	66.13	59.79	78.26	67.39
1.P+T-Syn-B	67.03	61.24	79.09	**68.51**
1.P+T-CUI+T-Syn	65.89	60.23	77.85	66.82
1.P+T-CUI+T-Syn-B	66.82	**61.28**	78.90	68.27

1.P: unigrams with POS, 2.P: bigrams with POS, T: Token, CUI: UMLS tag, Syn: expansion with synonyms, Syn-B: synonyms in break-down form. Best results per column are given in bold.

### Adding structural information

Table 6 shows the performance after structural information is added to unigrams with POS. These features produce a significant gain over the lexical and semantic features, achieving higher performance than the benchmark system: performance over structured abstracts is close to 80% f-score for 6-way classification, and close to 90% f-score for 5-way classification. For unstructured abstracts, using the *position* feature results in significantly improved performance over the use of lexical and semantic features alone. This indicates the importance of structural information to our task.

**Table 6:** F-scores using Structural Information.

Feature	6-way		5-way	
		S	U	S	U
1.P+Pst	73.26	**64.01**	83.64	**70.88**
1.P+Sec	79.22	–	88.88	–
1.P+Sec_M_	76.95	–	87.48	–
1.P+Pst+Sec	**79.67**	–	**89.19**	–
1.P+Pst+Sec_M_	78.45	–	88.55	–

1.P: Unigrams with POS, Pst: Position, Sec: Section heading, Sec_*M*_: Section heading with mapping. Best results per column are given in bold.

### Adding sequential information

For sequential information, we use previous sentences to inform the classifier. Our motivation is to measure whether explicitly adding sequential features is able to improve over the standard use of CRF. We combine this information with a basic set (B) of features, consisting of the following: unigrams with POS, position and section headings (for structured abstracts only). Since the labels of these sentences are not known, we follow two approaches:

• *Direct approach*: we use predictions of the labels of previous sentences by classifying them with a base learner. As features for the base classifier we also use the basic set B described above.

• *Indirect approach*: we use the features of the previous sentences as indirect indicators of their label. For simplicity, we use unigrams with POS, and add these features to the target sentence representation.

The results for the indirect approach are given in Table 7, together with the benchmark system. These features do not improve results over structured abstracts, but there are clear gains over the unstructured set, as large as 2.9% for 6-way classification. Even though sequential features may seem redundant when applying a sequential classifier, the results over unstructured data show that the extra information contributes to improved performance. Since unstructured abstracts cannot benefit from the main structural features, we conclude that the indirect approach is an effective way to close the gap in performance between the two types of abstracts.

**Table 7:** F-scores using 1 to 3 previous sentences (Indirect).

Feature	6-way		5-way	
		S	U	S	U
B+1 Prev. Sen.	**79.09**	65.06	**88.33**	71.80
B+2 Prev. Sen.	77.53	66.30	**88.33**	73.64
B+3 Prev. Sen.	76.75	**66.94**	88.03	**74.03**

B+Window	77.48	61.98	87.50	69.90

B: base features, Window: features in previous and posterior sentence. Best performance per column is given in bold.

Our next experiment uses the predicted tags of previous sentences as features for the target sentence (direct approach). We show the results using different window sizes in Table 8. In this case the performance gains over unstructured abstracts are not so clear, and the gains over structured abstracts are minimal. This suggests that the effort to build this architecture does not lead to improved performance due to the accumulation of errors, and that the indirect approach is a better strategy.

**Table 8:** F-scores using previous labels (Direct).

Feature	6-way		5-way	
		S	U	S	U
B+1 Prev. Label	79.85	63.64	89.24	71.15
B+3 Prev Labels	**80.88**	63.57	**89.32**	**71.54**
B+All Prev Labels	79.48	**64.66**	88.11	71.50

B: base features. Best performance per column is given in bold.

For this feature set we also present the results by class. In Table 9 we show the results for the best configurations from the direct and indirect experiments. The results illustrate that our *Outcome* and *Background* predictors are able to perform well, but the other classes exhibit lower f-score.

**Table 9:** F-scores per class from systems based on sequential features (applying the best configurations for each data subset).

Feature	6-way		5-way	
		S	U	S	U
Background	81.84	68.46	87.92	74.67
Intervention	20.25	12.68	48.08	21.39
Outcome	**92.32**	**72.94**	**96.03**	**80.51**
Population	56.25	39.80	63.88	43.15
Study Design	43.95	4.40	47.44	8.60
Other	69.98	24.28	–	–

Best performance per column is given in bold.

### Evaluation over external dataset

For external evaluation, we used the dataset from [18] to evaluate our classifiers. In this case the class *Study Design* is mapped into *Other*, and we build classifiers for 4-way and 5-way classification. The results are given in Table 10. The performance for the 5-way classifier is low, and only for structured abstracts are we able to reach 60% f-score. The results are better for 4-way classification (without *Other*), where the performances over structured and unstructured abstracts are similar, in the 70%-80% range.

**Table 10:** F-scores over dataset from [18].

Feature	5-way		4-way	
		S	U	S	U
Lexical & Structural				

1.P	55.12	37.10	76.90	76.32
1.P+Pst	57.80	38.53	78.04	72.82
1.P+Pst+Sec	62.83	–	83.81	–

Sequential (indirect)				

1.P+Pst+W	56.06	38.76	75.26	72.82
1.P+Pst+Sec+W	61.57	–	81.85	–
B+1 Prev. Sen.	62.36	**41.38**	**83.84**	75.20
B+2 Prev. Sen.	61.26	37.81	82.26	72.78
B+3 Prev. Sen.	60.16	37.81	82.20	75.27

Sequential (direct)				

B+1 Prev. Label	**63.15**	37.57	81.93	**79.21**
B+3 Prev. Label	**63.15**	36.39	77.72	76.98
B+All Prev. Labels	62.05	37.10	82.67	78.26

1.P: unigrams with POS, Pst: position, W: windowed features, Sec: section headings, B: base features. Best results per column are given in bold.

We also provide the results per class using our best classifiers; in Table 11 we see that our *Outcome* predictions perform well, but not those for other classes. These scores demonstrate significant disagreement between annotators for the classes *Intervention*, *Background*, and *Population*; further analysis would be required in order to find the reasons for these large discrepancies. [18] also reported difficulty in obtaining high agreement in the annotation, with *Outcome* being the most reliable class.

**Table 11:** F-scores per class over dataset from [18].

Feature	5-way		4-way	
		S	U	S	U
Background	56.18	15.67	77.27	37.50
Intervention	15.38	28.57	28.17	8.33
Outcome	**81.34**	**60.45**	**90.50**	**78.77**
Population	35.62	28.07	42.86	28.57
Other	46.32	15.77	–	–

For each of the two tasks (5-way, 6-way) the best feature set is applied. Best results per column are given in bold.

## Error analysis

In this section we analyse the confusion matrices of different experiments to identify the main sources of error. Table 12 shows the confusion matrix for our best-performing system over structured abstracts in cross-validation for 6-way classification. Each cell *i*, *j* in the matrix represents the number of cases where the gold-standard class *i* has been predicted as *j*. We can see that the main sources of error are: the prediction of Outcome instead of *Other* (155 errors, 15% of the total); and the prediction of *Other* for *Intervention* (132 errors, 13% of the total). The matrix also illustrates the difficulty of classifying *Intervention*, which only obtains 41 correct predictions.

**Table 12:** Confusion matrix over structured abstracts.

Class		Prediction					
				B	I	O	P	S	Ot
	B	561	4	43	8	2	51
G	I	27	41	48	60	5	132
o	O	6	1	2165	4	0	64
l	P	24	17	33	198	10	87
d	S	21	5	6	35	49	33
	Ot	63	24	155	30	8	754

B: Background, I: Intervention, O: Outcome, P: Population, S: Study Design, and Ot: Other.

The confusion matrix for unstructured abstracts is shown in Table 13. We see that there is a higher proportion of errors, and that the main errors are different than for structured abstracts. In unstructured abstracts, the classes *Background* and *Outcome* are the most confused: 496 errors (23% of the total) when *Outcome* is the goldstandard label, and 272 errors (13% of the total) when *Background* is the goldstandard label. This seems to indicate that the structure from the abstracts is particularly helpful in avoiding these types of errors. The label *Other* is also responsible for a high proportion of errors, being confused with *Outcome* 245 times (12% of the total).

**Table 13:** Confusion matrix over unstructured abstracts.

Class		Prediction					
				B	I	O	P	S	Ot
	B	1505	15	272	70	2	24
G	I	141	30	120	64	2	20
o	O	496	13	1722	18	0	34
l	P	161	24	73	158	1	26
d	S	36	3	7	26	2	10
	Ot	170	11	245	15	0	89

B: Background, I: Intervention, O: Outcome, P: Population, S: Study Design, and Ot: Other.

The results over our dataset show that the label *Other* is problematic. We manually analysed a sample of sentences annotated as *Other*, and found that a large proportion could be classified as special cases of the other labels. There were sentences referring to preparation for an intervention, or supporting treatments, that could be tagged as *Intervention*. Another group of sentences refers to the setting of the study (e.g., type of measurement applied), and these could be classified as a different label, or under *Background*. Finally, another set of sentences contain speculative content, which could be labelled as *Outcome*, or under a new label. Further study of these labels could provide insight for a more robust classifier. We also extracted the confusion matrix for the predictions when testing over the dataset from [18] (after training the classifier over our 1,000 abstracts). We show this information for 5-way classification in Table 14. We can see that most of the errors occur when our classifier predicts *Background* in place of the gold-standard class *Other* (74, 35% of the total). This indicates that the annotation of *Background* is particularly difficult, and the line between useful background knowledge and less relevant content is hard to define. The other main source of error is the prediction of *Outcome* in place of the gold-standard Other (53, 25% of the total). The matrix also shows the sparsity of the use of *Intervention* and *Population*, which only receive 8 and 13 correct predictions respectively.

**Table 14:** Confusion matrix when testing over dataset from [18] for structured abstracts.

Class		Prediction				
				B	I	O	P	Ot
	B	47	0	3	3	6
G	I	1	8	1	2	24
o	O	0	0	244	2	7
l	P	1	0	9	13	6
d	Ot	74	3	53	18	80

B: Background, I: Intervention, O: Outcome, P: Population, and Ot: Other.

Finally, Table 15 shows the confusion matrix for unstructured abstracts over the [18] dataset. As for structured abstracts, the main source of error is the prediction of *Background* in place of the goldstandard *Other* (134 errors, 54% of the total), and the prediction of *Outcome* instead of *Other* (71 errors, 29% of the total).

**Table 15:** Confusion matrix when testing over dataset from [18] for unstructured abstracts.

Class		Prediction				
				B	I	O	P	Ot
	B	21	1	1	1	3
G	I	3	3	3	1	1
o	O	3	0	112	0	2
l	P	4	1	6	4	3
d	Ot	134	0	71	8	12

B: Background, I: Intervention, O: Outcome, P: Population, and Ot: Other.

The error analysis of the predictions over the external dataset illustrates that the annotators seem to have followed different guidelines. There is a large set of *Other* sentences in the external dataset that are misclassified, and this could be due to the tendency in the external data to annotate only the most salient sentences as evidence for a particular label, ignoring supporting sentences.

## Conclusions

We have explored classification of sentences in abstracts with medical tags. Unlike previous work, we identify both irrelevant sentences and the semantic tags of relevant sentences using supervised techniques. We evaluated the performance of a variety of feature configurations over different sets of data, including an external corpus.

Our results for 5-way classification (which excludes the *Other* label) compare to the state of the art. The numbers are high for structured abstracts (89% f-score), but significantly lower for unstructured abstracts (74% f-score). However, for the latter we improve on the results of the benchmark system by 3.2% . The results for unstructured abstracts also demonstrate the difficulty of dealing with this kind of data, which has not been previously evaluated for this task. In the breakdown of the results per class, we see large differences in performance depending on the category, with *Outcome* showing strong performance, and *Intervention* and *Study Design* the weakest performance.

The 6-way classification task has not been previously explored using supervised approaches, and most work disregards irrelevant sentences. An exception is [7], which uses a small dataset for training an *Outcome* classifier, and utilises rule-based classifiers for the rest. In our experiments we can see that this is a very challenging task, particularly for unstructured abstracts, for which the f-score drops to 66.9%. Again, the application of our feature set is able to improve over the benchmark system, but the performance is much lower than for the 5-way task. We observed that sentences labelled as *Other* may have a large overlap with other labels, and further analysis of the annotation would be important in future work. Classification over the external dataset shows a drop in performance, and only for the category *Outcome* do we achieve good results. The cross-annotation of the other categories has proved problematic for the dataset from [18], and we need to further explore whether this is due to discrepancies in the annotations or the different domains of the training and test data.

With respect to the feature analysis, overall the best-performing features we used for our task were those based on unigrams, section headings, and sequential information from preceding sentences. Use of these features led to clear improvement over the simple BOW approach, and outperform feature sets used in previous work.

For future work, our aim is to improve our performance over unstructured abstracts with the aid of information from structured abstracts. We also plan to further analyse the annotated corpus, and in particular the sentences annotated as *Other*, to develop a more robust system. Finally, we plan to apply our classifier to an external application, such as improving performance of information retrieval against PICO criteria.

## Competing interests

The authors declare that they have no competing interests.
